# Microstructural characterization of random packings of cubic particles

**DOI:** 10.1038/srep35024

**Published:** 2016-10-11

**Authors:** Hessam Malmir, Muhammad Sahimi, M. Reza Rahimi Tabar

**Affiliations:** 1 Mork Family Department of Chemical Engineering and Materials Science, University of Southern California, Los Angeles, California 90089-1211, USA; 2 Department of Physics, Sharif University of Technology, Tehran 11365-9161, Iran

## Abstract

Understanding the properties of random packings of solid objects is of critical importance to a wide variety of fundamental scientific and practical problems. The great majority of the previous works focused, however, on packings of spherical and sphere-like particles. We report the first detailed simulation and characterization of packings of non-overlapping cubic particles. Such packings arise in a variety of problems, ranging from biological materials, to colloids and fabrication of porous scaffolds using salt powders. In addition, packing of cubic salt crystals arise in various problems involving preservation of pavements, paintings, and historical monuments, mineral-fluid interactions, CO2 sequestration in rock, and intrusion of groundwater aquifers by saline water. Not much is known, however, about the structure and statistical descriptors of such packings. We have developed a version of the random sequential addition algorithm to generate such packings, and have computed a variety of microstructural descriptors, including the radial distribution function, two-point probability function, orientational correlation function, specific surface, and mean chord length, and have studied the effect of finite system size and porosity on such characteristics. The results indicate the existence of both spatial and orientational long-range order in the packing, which is more distinctive for higher packing densities. The maximum packing fraction is about 0.57.

Properties of packings of non-overlapping particles have been of fundamental and practical interests for decades, and have been studied intensively. At the fundamental level such packings have been used as conceptual models to study and understand the structure of liquids, glassy and crystal states of matter^1^,^2^,^3^, granular media^4^, and heterogeneous materials^3^,^5^,^6^. From a practical view point, packings of particles are relevant to powders^7^, cell membranes^8^, thin films^9^, colloidal dispersion^10^, and composite materials^3^,^5^,^6^.

The great majority of the previous studies of packings of non-overlapping particles were devoted to those in which the particles were spherical, with relatively limited studies of elliptical particles^11^,^12^,^13^,^14^,^15^. More recently, attention has been focused on packings of particles with more complex shapes, including disks^16^,^17^, hard rectangles^18^, Platonic and Archimedean solids^19^,^20^, tetrahedral^21^,^22^,^23^, and other types^24^. Many of the issues relevant to packings of solid objects have been reviewed by Torquato and Stillinger^25^.

In this paper we report on the results of extensive computer simulation of microstructural characterization of an important, yet unexplored type of packings, namely, those that consist of non-overlapping *cubic* particles. Such packings are encountered in biological materials, colloids^26^ and other types of systems of scientific importance. An important practical example is evaporation of saline water. As evaporation proceeds, salt crystallizes and precipitates on the surface of the system in which the water is flowing, giving rise to a packing of cubic salt crystals that damages the surface. Understanding this phenomenon and how the packing changes the morphology of the system in which salt has precipitated are of fundamental importance to preservation of pavements, paintings, and historical monuments, mineral-fluid interactions, CO_2_ sequestration in rock, and intrusion of groundwater aquifers by saline water, as the world faces increasing difficulty in obtaining the drinking water that it needs^27^. New methods for fabrication of scaffolds involve salt leaching in salt powder and the structure of the scaffolds depends critically on the microstructure of the salt powder^28^,^29^.

The first step toward understanding the properties of random packings of cubic particles is generating a model for them. Development of an efficient algorithm for disordered packings of cubes, particularly disordered jammed packings, has been traditionally considered as a *hard problem.* We have developed a version of the random sequential addition (RSA) algorithm to generate such packings. The reason for using the RSA algorithm is that other algorithms, such as various molecular dynamics and Monte Carlo methods for hard-particle packings are not applicable to cubic particles, because the overlap potential functions cannot be constructed for particles with non-smooth shapes, including all the Platonic and Archimedean solids.

## Results

**Figure 1:**
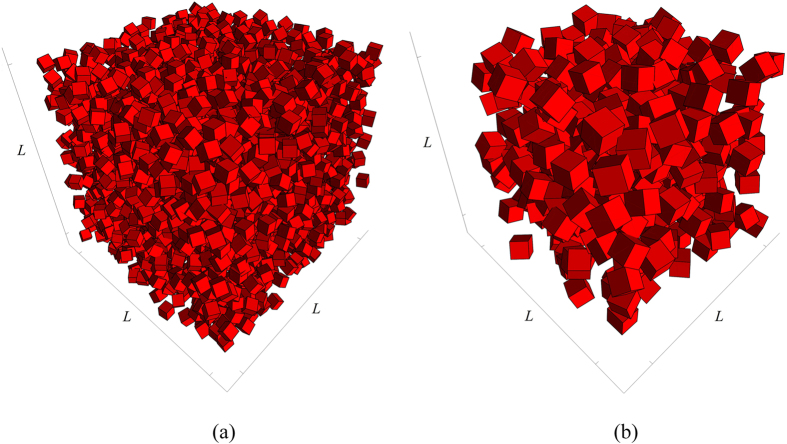
Packings of non-overlapping equal-size cubic particles with (**a**) *N* = 2600 and *d*_0_ = 0.05*L* (**b**) *N* = 400 and *d*_0_ = 0.1*L*, with *N* being the number of particles.

We assume that all the particles have the same size with length of their edge being *d*_0_. We refer to the pore space and solid particles as phases 1 and 2, respectively. The structure of any random packing of non-overlapping particles depends on the packing density *ϕ*_2_ and the porosity *ϕ*_1_. Figure 1 presents two random packings with the same particle density *ϕ*_2_ = 0.3, but with different particle sizes of *d*_0_ = 0.05*L* and *d*_0_ = 0.1*L*, with the size of the simulation cell being *L* × *L* × *L*. For a fixed packing density *ϕ*_2_, smaller particles lead to better structured configurations. This is due to the finite system size that are particularly important for large particles.

In order to analyze the statistical properties of the random packings, we have computed several of their most important microstructural descriptors, including the radial distribution function *g*(*r*), the two-point probability function *S*_2_(*r*), the face-normal correlation function *C*_FN_(*r*), the specific surface *s*, and the mean chord length *l*_*C*_. For each case we have studied the effect of two important factors, namely, the size of the particles and the porosity of the packings.

### Microstructural Descriptors

**Figure 2:**
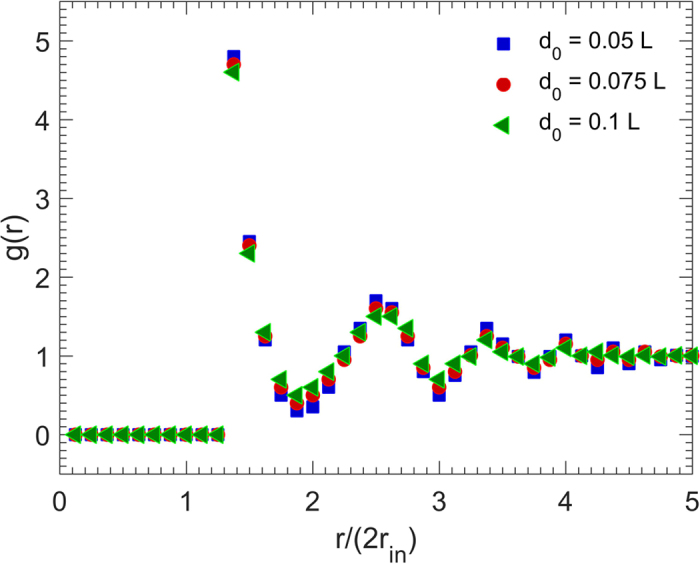
Dependence on the particle size of the radial distribution function *g*(*r*) of the packings with the packing density *ϕ*_2_ = 0.45. *r*_in_ is the radius of the cube insphere.

The radial distribution function *g*(*r*) is the probability of finding a particle at a distance of *r* away from a given reference particle, and describes how density of a system varies as a function of *r*. Figure 2 presents *g*(*r*) for three packings with three particle sizes. The packing density *ϕ*_2_ = 0.45 is fixed for all the three packings. For all the cube sizes, *g*(*r*) has its first peak at distance *r* ≈ 2.8*r*_in_ = 1.4*d*_0_, where *r*_in_ = *d*_0_/2 is the cube insphere radius, in agreement with theoretical expectation. Furthermore, the fluctuation of *g*(*r*) beyond the first peak indicates an order-on-average of the disordered packing. This behavior is similar to the predictions of the analytic solutions of the Percus-Yevick equation of fluids and experimental data for hard spheres^2^,^30^. The oscillations also indicate long-range order in the packing. The evidence for order is that, in its absence *g*(*r*) decays to unity very rapidly. In other words, the deviations of *g*(*r*) from unity signify the degree of spatial correlation between the particles, with unity corresponding to no spatial correlation. Note that, the smaller the particles’ size, the larger are the oscillations in *g*(*r*), demonstrating that the crystallinity of the packing is improved for larger number of the particles of smaller sizes. Thus, such an effect represents finite-size effect for larger particles.

**Figure 3:**
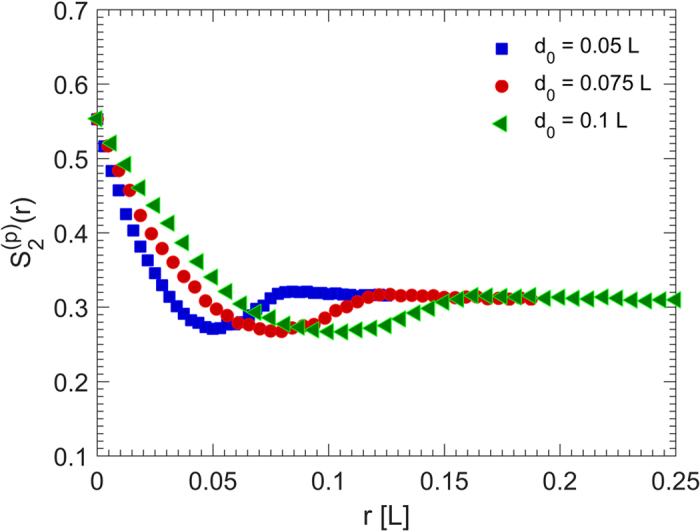
Dependence on the particle size of the two-point probability function 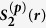 for the pore phase of the packings with the packing density *ϕ*_2_  = 0.45. The size of the simulation cell is *L* × *L* × *L*.

The two-point probability function 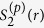 is the probability that two points separated by a distance *r* are both in the pore space (a similar function can be defined for the solid phase). The computed 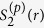 for the same three packings of Fig. 2 are presented in Fig. 3. The porosity of the packings is, 
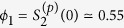
. Furthermore, 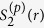 approaches 0.31 for large *r* since, theoretically, 
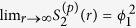
. As illustrated in Fig. 3, although the initial and final values (*ϕ*_1_ and 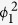, respectively) are the same, the slope of the two-point probability function, as well as its rate of approach to 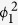 are different for various particle sizes. Furthermore, the minima of the curves occur at distances equal to the corresponding particle sizes, implying that the probability of finding two end points of a line segment *r* follows the same behavior, but the rate of approach depends on the particles’ size. This feature affects the distance between particles’ surfaces, as well as the ratio surface/volume of the packing.

**Figure 4:**
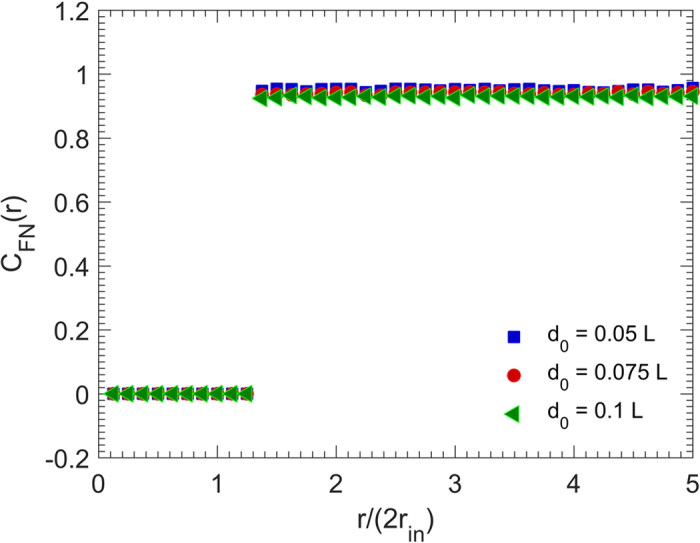
Dependence on the particle size of the face-normal correlation function *C*_FN_(*r*) of the packings with the packing density *ϕ*_2_ = 0.45, where *r*_in_ is the radius of the cubes’ insphere.

We also computed the face-normal correlation function *C*_FN_(*r*), representing an orientational correlation between different particles’ faces. Figure 4 presents the results. When the orientational long-range order is present in the system, the particles have many face-to-face contacts and the largest angle between two normal vectors to the faces of the cubes will be *π*. Hence, *C*_FN_(*r*) must be close to unity even at far distances. Figure 4 indicates that *C*_FN_(*r*) is indeed in the interval (0.9, 1), and close to unity for all radial distances. Figure 4 also indicates that the size of the particles does not strongly affect this important feature of *C*_FN_, and that the RSA packings of cubic particles possess orientational long-range order.

Two key characteristics of packings that are useful for estimating their flow and transport properties are the specific surface *s* and mean cord length *l*_*C*_. More generally, one may also define a cord length distribution function. Table 1 presents the two quantities for the three packings of Fig. 2. Since the two parameters are directly calculated from the slope of the two-point probability function 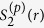, we find that the slope of the function and its approach to 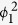 are dependent on the size of the particles and, therefore, for a fixed density, packings of smaller particles have larger specific surfaces (the interfacial area per unit volume), which is the basis for fabrication of porous materials with large internal surface. The pore space of the same packings also have smaller chord lengths. We find that the relations between the two characteristics, the particles’ size and the dimension of the system, are approximately, *s*(*d*_0_) ≈ −516.74*d*_0_/*L* + 71.66 and *l*_*C*_(*d*_0_) ≈ 1.16*d*_0_/*L* − 0.01. Such correlations are useful for the calculation of the various flow and transport properties of porous media^31^,^32^.

### Effect of the Porosity

**Figure 5:**
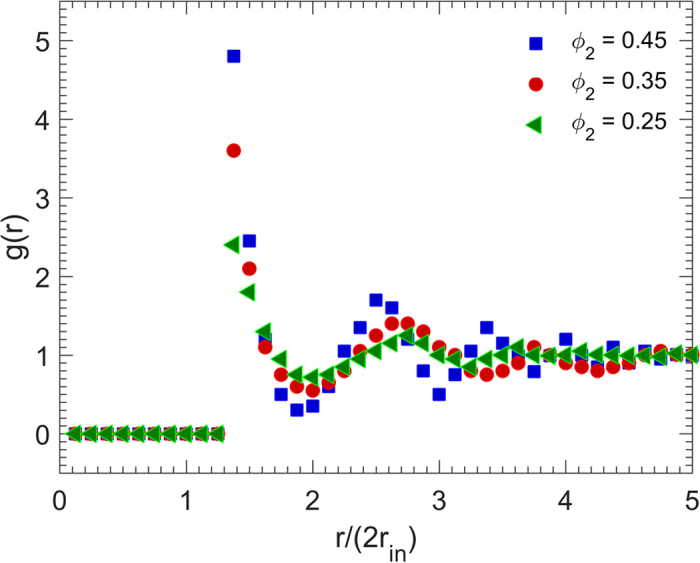
Dependence of the radial distribution function *g*(*r*) on the packing density *ϕ*_2_. *r*_in_ is the radius of the cubes’ insphere, and the size of the particles is *d*_0_ = 0.05*L*.

The radial distribution functions *g*(*r*) for the packings with fixed particle size *d*_0_ = 0.05*L*, but various packing densities are presented in Fig. 5. As the figure indicates, both the first peak and the oscillations of *g*(*r*) around unity vary with the packing density. Although the first peak of *g*(*r*) for all the packing densities occurs at *r* ≈ 2.8*r*_in_ = 1.4*d*_0_, its magnitude varies from 4.8 for *ϕ*_2_ = 0.45 to 3.6 for *ϕ*_2_ = 0.35 and 2.4 for *ϕ*_2_ = 0.25. This is expected as the number of the nearest neighbors of a cubic particle is larger for higher packing densities.

In addition, it is well-known that the mean number *Z* of the nearest-neighbors of a given particle is given by


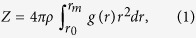


where *r*_0_ is the rightmost position starting from *r* = 0 at which *g*(*r*) = 0, *r*_*m*_ is the position of the first minimum after the first peak, and *ρ* is the number density of particles. The results are *Z* = 4.31, 3.46 and 2.4 for *ϕ*_2_ = 0.45, 0.35, and 0.25, respectively, and agree with our computations.

Moreover, the distance between the first and second peaks of *g*(*r*) is smaller for higher packing densities. Besides, the higher the packing density, the more strongly is the fluctuating behavior and, hence, the stronger is the long-range order. In fact, in the absence of long-range order, the cubic particles are mutually far from one another with no spatial correlation between them, leading to the rapid decay of the radial distribution function to unity. Hence, since the oscillations of *g*(*r*) around unity is much larger for higher packing densities, we deduce that the denser RSA packings of cubic particles exhibit better spatial long-range order.

**Figure 6:**
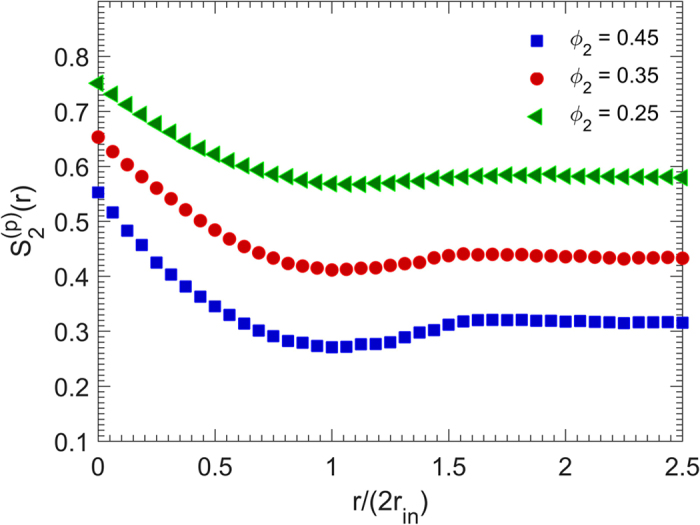
Dependence of the two-point probability function 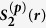 on the packing density *ϕ*_2_ . *r*_in_ is the radius of the cubes’ insphere, and the particles’ size is *d*_0_ = 0.05*L*.

The two-point probability function 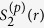 of the pore phase of the same packings are shown in Fig. 6. The porosities are 
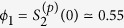
, 0.65 and 0.75 for the aforementioned packing densities, and 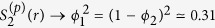, 0.43 and 0.57, as the radial distance *r* increases. Furthermore, as *ϕ*_2_ decreases, i.e. as the pore sizes increase, 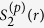 becomes a weaker function of the distance, since the pore space has been enlarged and, therefore, the probability that two points separated by a distance *r* in the pore space becomes more or less independent of the distance. The slope of 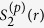 with respect to *r* is greater for higher density, which influences the specific surface and the mean distance between the particles’ surfaces, i.e. the mean chord length.

**Figure 7:**
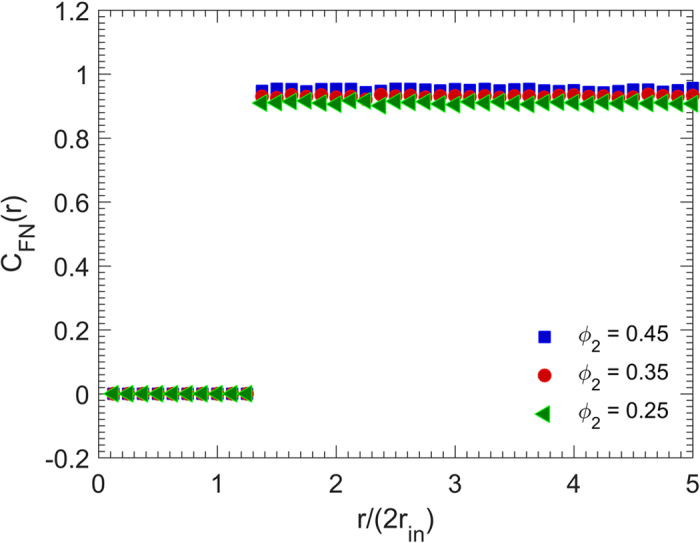
Dependence of the face-normal correlation function *C*_FN_ on the packing density *ϕ*_2_. *r*_in_ is the radius of the cubes’ insphere, and the particles’ size is *d*_0_ = 0.05*L*.

The corresponding face-normal correlation functions *C*_FN_(*r*) for the same packings are exhibited in Fig. 7. As shown, the higher the packing density, the closer to unity is the orientational correlation function *C*_FN_(*r*). Furthermore, independence of this feature from the radial distance *r* indicates the existence of orientational long-range order. We therefore conclude that the packing of cubic particles have both spatial and orientational long-range order, particularly at higher packing densities.

We also computed the specific surface *s* and mean cord length *l*_*C*_ for the pore space of the same packings. The results are listed in Table 2. The pore space of the packings with higher densities has larger specific surfaces, but smaller chord lengths. In addition to physical ground, this was expected from the slope of 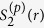 with respect to *r* at *r* = 0 for various packing densities; Fig. 6. It means that packings of cubic particles with higher densities have greater specific surfaces, but smaller distances between the surfaces of the cubic particles. The computed results can be approximated by *s*(*ϕ*_2_) ≈ 107.87*ϕ*_2_ − 2.34 and *l*_*C*_(*ϕ*_2_) ≈ −0.36*ϕ*_2_ + 0.2.

## Discussion

Random packings of non-overlapping equal-sized cubic particles possess both spatial and orientational long-range order, particularly at high packing densities. As long as the densest achievable packing is 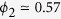, the packings with a fixed density but smaller particle sizes have larger specific surfaces and smaller chord lengths in the pore space, which is the same for packings with fixed particle sizes but higher densities. Such insights between the characteristic functions and the size of the particles and the packing density may be used for fabrication of porous materials with large internal surface. Indeed, such efforts are currently underway; the results will be reported in the near future.

Although there is currently no detailed simulations of statistical descriptors for packing of cubic particles, our computed maximum packing fraction may be compared with the work of Baker and Kudrolli^20^ and that of Agarwal and Escobedo^33^. The maximum packing fractions in these works is 0.45 for the isotropic phase and 0.57 for the cubatic phase (mesophase or liquid-crystal state). Packing fractions greater than 0.57 represent the crystal phase, which cannot be achieved by random close packings. Hence, the maximum packing fraction computed in our work, 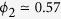, bears close resemblance to that of the cubatic phase and, thus, exhibits the mesophase behavior. Furthermore, the statistical descriptors analyzed in this work are mostly related to the packing fractions in the isotropic phase (below *ϕ*_2_ = 0.45). As we demonstrated, by increasing the packing fraction to the limits of this phase, *ϕ*_2_ → 0.45, the long-range order is better developed, and the packing demonstrates mesophase (liquid-crystal) behavior.

Further comparison of our results with those of the dense packings of other Platonic solids^19^,^20^, and in particular tetrahedral particles, indicates that random packings of cubic particles have better-structured configurations. In particular, dense packings of tetrahedral particles^19^ possess short-range order. Although the radial distribution function for such packings exhibits behavior similar to that of packings of cubic particles but with faster decay to unity, their orientational correlation function indicates face-to-face contacts between only the neighboring particles, implying short-range orientational correlations. This is in contrast with the random packings studied here that exhibit both spatial and orientational long-range order.

## Methods

Packings of spherical and sphere-like particles maybe generated by a variety of algorithms, including the random sequential addition (RSA) algorithm^12^,^34^,^35^,^36^,^37^,^38^, particle-growth molecular dynamics method^16^,^39^,^40^,^41^ and the Monte Carlo schemes^17^,^18^,^42^,^43^. We have developed an algorithm for generating packings of cubic particles based on the RSA method, which we describe next. Another efficient algorithm was suggested by Munjiza and Latham^44^.

### Algorithm for generating the packings

The RSA is a process for generating disordered packings of *d*-dimensional particles in 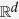. To use the algorithm for generating random packing of cubic particles, we begin with a large, empty region of volume *V* in 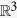, generate cubic particles with randomly-selected positions and orientations, and place them sequentially in the volume. The deposition is subject to the non-overlapping constraint, so that no newly inserted particle can overlap with any existing ones. The addition process can be stopped at any step. The computational details of the algorithm are as follows.

**Step 1**. Specify the total number of cubic particles, *N*, and the cubes’ length *d*_0_, along with the size of the simulation cell *L*_*x*_ × *L*_*y*_ × *L*_*z*_.

**Step 2**. Generate three random numbers *x*_*c*_ ∈ (0, *L*_*x*_), *y*_*c*_ ∈ (0, *L*_*y*_) and *z*_*c*_ ∈ (0, *L*_*z*_) for the center of a new cubic particle.

**Step 3**. Generate two random numbers *u* ∈ [−1, 1] and *ϕ* ∈ [0, 2*π*) for the normal vector ***n*** of the upper face of the cubes. The normal vector is expressed by


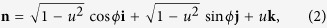


where **i**, **j**, **k** are the three unit vectors in Cartesian coordinates (*x*, *y*, *z*).

**Step 4**. Determine the matrix **R** that rotates the unit vector **k** into the unit vector **n** through


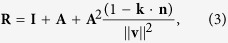


where **I** is the identity matrix, and the unit vector **v** = (*v*_1_, *v*_2_, *v*_3_) is defined as **k** × **n**. Furthermore,


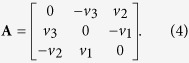


It should be noted that if **n** = **k**, then **R** = **I**, and if **n** = −**k**, we have, **R** = −**I**.

**Step 5**. The coordinates of the cube’s eight vertices, **V**_*i*_ for *i* = [1, 2, ···, 8] are obtained by


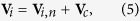


where **V**_*c*_ is the coordinate vector of the cube’s center, and **V**_*i*,*n*_ = **RW**_*i*_, in which **W**_1_ = (−*d*_0_/2, −*d*_0_/2, −*d*_0_/2), ···, **W**_8_ = (*d*_0_/2, *d*_0_/2, *d*_0_/2).

**Step 6**. Check if all the cube’s vertices are outside the previously-inserted cubes. If so, the particle is accepted, and *n* → *n* + 1, where *n* is the number of generated and accepted cubical particles. If (*n* ≤ *N*), go to **Step 2** or, else, terminate the simulation.

It is of noteworthy that the non-overlapping constraint (NOC) in step 6 can be replaced by any other constraint. Various NOCs result in diverse packing configurations and microstructural properties. For example, one may define the NOC as the distance between a new cube’s vertices and those of the previously-inserted cubes that must be greater than or equal to 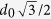. Such a constraint results in smaller packing density when compared to the aforementioned constraint.

### Microstructural descriptors

As already mentioned, we compute several microstructural descriptors of the packings that are as follows.

#### Radial distribution function

The most basic statistical descriptor for isotropic systems is the radial distribution function *g*(*r*), where *g*(*r*)*r*^2^*dr* is proportional to the conditional probability that a particle’s centroid is found in a spherical shell of thickness *dr* at a radial distance *r* from another particle’s centroid at the origin. When there is no long-range order in the system, *g*(*r*) decays to unity very rapidly. For crystalline and polycrystalline structures, however, in which remote portions of the same sample exhibit correlated behavior, *g*(*r*) exhibits pronounced oscillations around unity.

#### Two-point probability function

The two-point correlation function 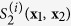 for phase *i* of a multiphase system, defined as


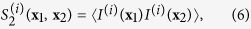


is one of the most important statistical descriptors of random media. It represents the probability of finding two randomly-selected points **x**_1_ and **x**_2_ in phase *i*. The indicator function *I*^(*i*)^(**x**) = 1 if **x** belongs to phase *i* and is zero otherwise. For statistically homogeneous and isotropic media, 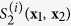 depends only on the distances, i.e.


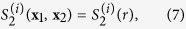


where *r* is the distance between **x**_1_ and **x**_2_. One also has, 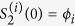, in which *ϕ*_*i*_ is the volume fraction of phase *i*. In addition, 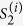 must satisfy, 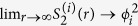. Finally, for two-phase disordered media one has, 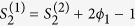. Note that there are certain relations between 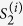 and other microstructural descriptors^3^,^5^, so that knowledge about 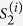 leads directly to information about such descriptors.

#### Face-normal correlation function

An important statistical descriptor for packings of nonspherical particles is the face-normal correlation function C_FN_(r), defined as the average of the largest negative value of the inner product of two face normals of a pair of cubes *p* and *q*, separated by a distance *r*:


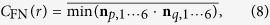


Where the overline represents the average. The face-normal correlation function measures the extent to which a cube’s orientation affects the orientation of another cube at a different position.

#### Specific surface and mean chord length

The specific surface *s*_*i*_, defined as the interfacial area per unit volume, represents global information about the internal surface of the *i*th phase. It can be shown that for *d*-dimensional isotropic porous media *s*_*i*_ can be obtained using the first derivative of the two-point probability function 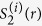 through


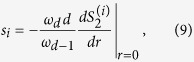


where, *ω*_*d*_ = *π*^*d*/2^/Γ(1 + *d*/2) is the *d*-dimensional volume of a sphere of unit radius. The formula is applicable to anisotropic media as well, after angular averaging.

It is straightforward to show that, for statistically isotropic systems of arbitrary microstructure, the mean chord length 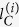, defined as the mean probability of finding a chord of length between *z* and *z* + *dz* in phase *i*, is related to the slope of the probability function 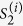 at the origin via


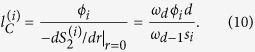


## Additional Information

**How to cite this article**: Malmir, H. *et al.* Microstructural characterization of random packings of cubic particles. *Sci. Rep.* **6**, 35024; doi: 10.1038/srep35024 (2016).

## Figures and Tables

**Table 1:** Dependence on the particles’ size of the specific surface *s* and mean chord length *l*_*C*_ for the pore phase of the packings with packing density *ϕ*_2_ = 0.45.

	*d*_0_ = 0.05*L*	*d*_0_ = 0.075*L*	*d*_0_ = 0.1*L*
*s (L*^−1^)	46.86	30.83	21.02
*l*_*C*_ (*L*)	4.72 × 10^−2^	7.17 × 10^−2^	10.53 × 10^−2^

**Table 2:** Dependence on the packing density *ϕ*_2_ of the specific surface *s* and mean chord length *l*_*C*_ of the pore phase of the packings with the same particle sizes, *d*_0_ = 0.05*L*.

	*ϕ*_2_ = 0.45	*ϕ*_2_ = 0.35	*ϕ*_2_ = 0.25
*s (L*^−1^)	46.86	34.11	25.28
*l*_*C*_ (*L*)	4.72 × 10^−2^	7.66 × 10^−2^	11.88 × 10^−2^
